# Accelerating Solvent Dynamics with Replica Exchange for Improved Free Energy Sampling

**DOI:** 10.1021/acs.jctc.3c00786

**Published:** 2023-10-21

**Authors:** Robert Darkins, Dorothy M. Duffy, Ian J. Ford

**Affiliations:** Department of Physics and Astronomy, University College London, Gower Street, London WC1E 6BT, U.K.

## Abstract

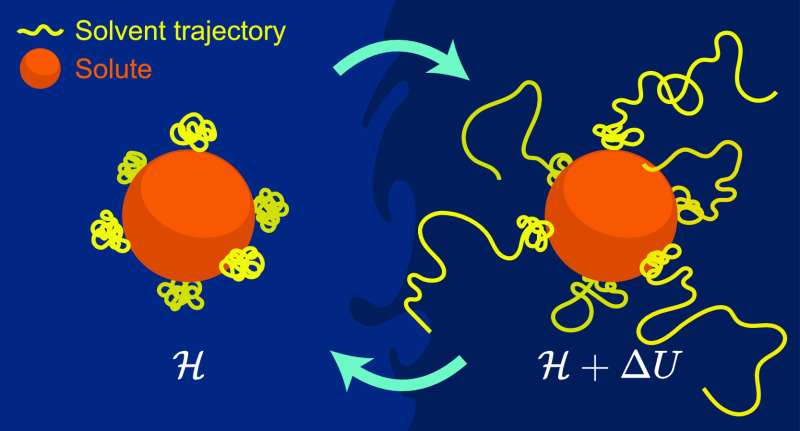

Molecular reactions in solution typically involve solvent exchange; for example, a surface must partly desolvate for a molecule to adsorb onto it. When these reactions are simulated, slow solvent dynamics can limit the sampling of configurations and reduce the accuracy of free energy estimates. Here, we combine Hamiltonian replica exchange (HREX) with well-tempered metadynamics (WTMD) to accelerate the sampling of solvent configurations orthogonal to the collective variable space. We compute the formation free energy of a carbonate vacancy in the calcite–water interface and find that the combination of WTMD with HREX significantly improves the sampling relative to WTMD without HREX.

## 1 Introduction

Free energy methods are routinely used to study the thermodynamics of molecular reactions in solution. However, strong solute–solvent binding can lead to slow solvent exchange, and this can diminish the accuracy of free energy estimates by (1) limiting the sampling of solvent configurations and (2) restricting access to specific states due to the lag between solute and solvent dynamics. This issue frequently arises when simulating adsorption on ionic surfaces, and it can be addressed by promoting faster exchange of solvent molecules within solvation shells.^1^

A popular approach to determine reaction thermodynamics in solution is to use a biasing method like metadynamics^2^ where the free energy surface is computed as a function of a small number of collective variables (CVs). The CVs represent the progress of the reaction in a low-dimensional space. The problem of slow solvent dynamics is usually addressed by including the solvent coordination numbers of relevant solutes as CVs.^3−7^ However, the cost of this approach means that it can only be applied to accelerate the solvent exchange of one or two solute atoms. Moreover, since the dynamics of coordination numbers are not strictly Markovian, this approach could produce dubious free energies, especially when applied to solutes with large coordination numbers and complex solvation shells.

Rather than expanding the CV space to include a measure of the solvent structure, we propose combining metadynamics with a replica exchange method to accelerate the sampling of solvent coordinates orthogonal to the CV space. Replica exchange methods improve ergodicity by allowing the atomic configuration to randomly walk through different ensembles in which barriers are reduced either by elevated temperatures, as in parallel tempering (PT),^8^ or by modified potential energy surfaces, as in Hamiltonian replica exchange (HREX).^9^ These replica exchange methods have previously been used to mitigate the effects of strong solvation; for example, PT has been used to accelerate surface dehydration during the adsorption of a protein,^10^ while HREX has been used to scale the hydrophobicity of proteins in both implicit^9^ and explicit^11,12^ solvents. These methods have also been combined with metadynamics to enhance configurational sampling, although current applications primarily focus on biomacromolecules.^13−20^

In this paper, we combine HREX with well-tempered metadynamics (WTMD), although other free energy methods may be substituted, and modify the potential energy surface to accelerate the sampling of solvent configurations. We apply this method to compute the formation free energy of a carbonate vacancy in the calcite–water interface and compare it to the results obtained using WTMD without HREX.

## 2 Method

### 2.1 WTMD–HREX

The aim is to use WTMD to compute the free energy surface *F*(ξ) as a function of the CVs ξ for a Hamiltonian  while using HREX to accelerate the sampling of slow coordinates orthogonal to the CVs.

**Figure 1:**
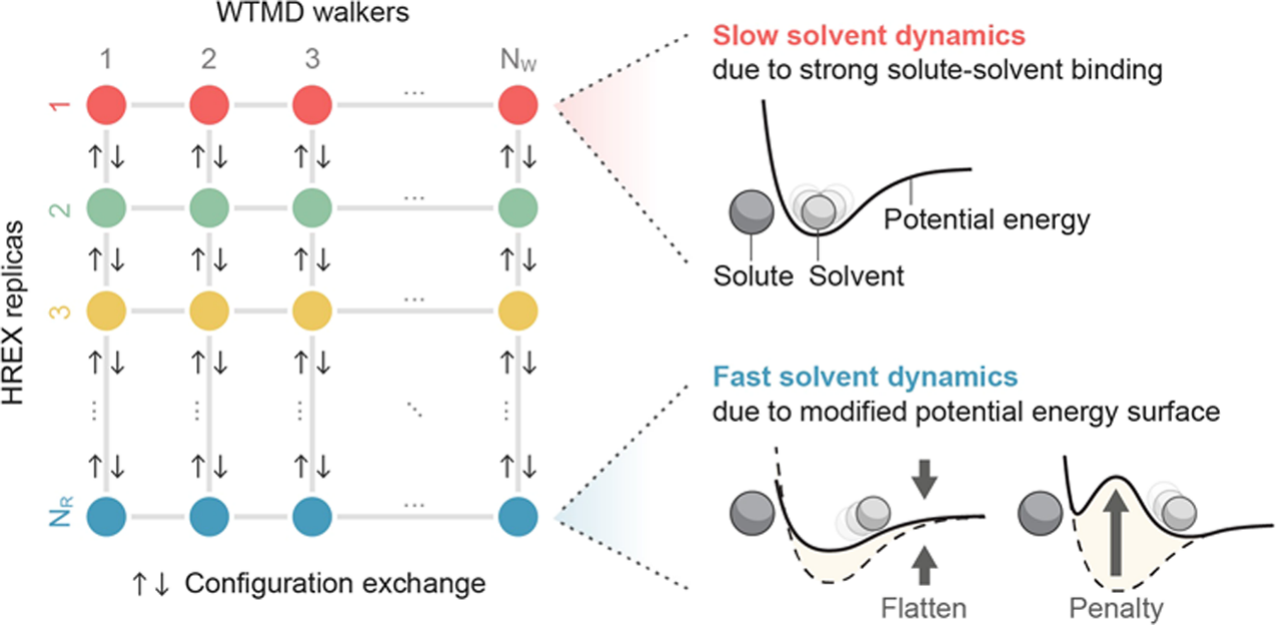
WTMD–HREX involves *N*_R_ × *N*_W_ simulations (circles) composed of *N*_R_ replicas and *N*_W_ walkers per replica. Replica one (red) samples the intended potential energy surface, while the other replicas have increasingly modified potential energy surfaces that promote faster solvent dynamics. The potential energy surfaces are modified either by flattening selected interactions or by adding energy penalties to repel the solvent. Neighboring replicas randomly exchange configurations according to the Metropolis criterion, boosting the ergodicity of replica one. Multiple walkers cooperate to accelerate sampling and convergence.

Multiple molecular dynamics simulations are run concurrently. The simulations are divided into *N*_R_ replicas and *N*_W_ metadynamics walkers per replica, forming a grid of *N*_R_ × *N*_W_ simulations (Figure 1). Each simulation (*i*, *j*) is denoted by its replica *i* and walker *j*.

The *N*_W_ walkers assigned to replica *i* share the time-dependent Hamiltonian
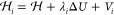
1where the alchemical parameters λ_*i*_ increase monotonically from λ_1_ = 0 to λ_N_R__ = 1, Δ*U* is a potential energy modification, prescribed below, and *V*_*i*_ is the multiple-walkers^21^ well-tempered^22^ metadynamics bias potential, which is built up iteratively at time intervals of τ_deposit_. On the *n*th iteration,
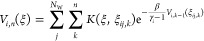
2where β = 1/(*k*_B_*T*), *k*_*B*_ is the Boltzmann constant, *T* is the temperature, γ_*i*_ > 1 are the bias factors, ξ_*ij*,*k*_ are the CVs of simulation (*i*, *j*) at iteration *k*, and the Gaussian kernel is
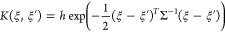
3where *h* is the initial Gaussian height, Σ_*ab*_ = σ_*a*_^2^δ_*ab*_ is a diagonal matrix, and σ_*a*_^2^ is the variance of the Gaussian in the *a*-th CV.

For each simulation (*i*, *j*), the configuration *X*_*ij*_, which consists of the positions and velocities of all atoms, is periodically exchanged with its neighboring replica *X*_(*i*+1)*j*_ with probability
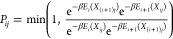
4where *E*_*i*_ = λ_*i*_Δ*U* + *V*_*i*_. When *i* is even (odd), exchange attempts are made between replicas *i* and *i* + 1 at times corresponding to even (odd) multiples of τ_swap_. This means that each replica *i* attempts to exchange with replicas *i* – 1 and *i* + 1 on alternating iterations, which is an efficient exchange scheme.^23^

Finally, the free energy surface for replica *i* is
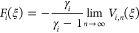
5modulo a constant, and the free energy surface being sought is *F*(ξ) = *F*_1_(ξ).

### 2.2 Δ*U* for Fast Solvent Dynamics

To accelerate the exchange of solvent molecules within the solvation shells of certain solutes, we propose a potential energy modification of the form
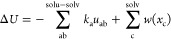
6where *k*_a_ ∈ [0, 1) is a constant associated with atom a, *u*_ab_ is the interaction energy between atoms a and b, and *w* is an energy penalty function that acts on the position *x*_c_ of atom c. The first sum is over all pairwise interactions between atoms a in the solutes and atoms b in the solvent, and the second sum is over all atoms c in the solvent.

The purpose of the first term is to weaken the interaction between a particular selection of solute atoms and the solvent. By subtracting *k*_a_*u*_ab_ from Δ*U*, the interaction energy between atoms a and b in replica *N*_R_ becomes (1 – *k*_a_)*u*_ab_. For most solute–solvent interactions, *k*_a_ will be zero, and no change to the interaction will be made. But for solutes that have strong solvation shells and that take part in the reaction under study, a choice of *k*_a_ > 0 will flatten the potential energy surface and promote faster solvent exchange (Figure 1). For example, *k*_a_ = 0.1 will make atom a bind 10% more weakly with the solvent.

In many cases, the first term in eq 6 will suffice. But if the solvent continues to exhibit slow exchange in a particular region, such as a nook of a surface, then the second term can be included to repel the solvent from that region, as depicted in Figure 1. We choose *w* to be a Gaussian
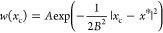
7where *A* is the height, *B* is the width, and x* is the position of the energy penalty. Multiple instances of eq 7 may be added to repel the solvent from multiple regions.

### 2.3 Practical Guidelines

Here is a summary of the practical steps required to apply our method:

1. Install a molecular dynamics code that supports WTMD–HREX. We used LAMMPS^24^ and a custom LAMMPS package incorporating WTMD–HREX. We have made this package available to the public as an open-source release.^25^
2. Identify the atoms with slowly exchanging solvation shells that are relevant to the reaction, e.g., by recording solvent residence times.^26^
3. Determine a suitable modification Δ*U* by weakening the relevant solute–solvent interactions so that the solvent molecules exchange on a sub-100 ps time scale. This is done through trial and error by increasing *k*_a_ and running short simulations to check whether the solvent exchanges quickly enough.
4. Determine whether an energy penalty is needed by running a brief simulation with the modified Hamiltonian . Drag the CV (e.g., adsorbate position) into the reaction state (e.g., a vacancy) that is impeded by the solvent in the unmodified system. The dragging force should be at least as large as the forces typical of the WTMD bias potential (∼10 *k*_B_*T*/Å). If access to the state is still impeded by the solvent, an energy penalty should be added to exclude the solvent from that region.
5. Choose the number of replicas *N*_R_. In standard HREX, an optimal choice for *N*_R_ is characterized by an acceptance probability of 39% for exchange attempts, although deviations from this value incur only small costs.^27^ However, 39% should be considered an upper bound when combining HREX with metadynamics since it may be more efficient to use fewer replicas in favor of additional walkers.
6. Perform a preliminary simulation to estimate well depths in each replica so that appropriate bias factors γ_*i*_ can be chosen. It will likely suffice to pick γ_1_ and γ_N_R__ and interpolate linearly between them.
7. Run the simulation until the free energy surface converges.
8. Verify that the continuous trajectories walking through replica space display transitions between the different states in the CV space. This behavior ensures an equilibrium population of trajectories. If transitions are not observed, the solvent may still be impeding sampling in the highest-order replica, and so, the modification Δ*U* may need revising.

## 3 Application

We used our method to compute the formation free energy of a CO_3_^2–^ vacancy at the calcite–water interface. This interface is notorious for its strongly hydrated Ca^2+^ ions and associated slow water exchange.^26^ A CO_3_^2–^ vacancy is particularly challenging because it is formed by five of these strongly hydrated Ca^2+^ ions (four within the surface plane and one below), and there is a cavity for the water to get trapped in. Tackling this vacancy problem will demonstrate that the method is capable of solving the more common but less demanding adsorption problems involving terraces, steps, and kinks.

### 3.1 Simulation Details

The simulation consisted of a calcite slab with a pair of {101̅4} surfaces cleaved normal to the *z* axis and separated by water in a periodic supercell. The *z* coordinate of a CO_3_^2–^ ion in the upward-facing surface, referred to as the adsorbate hereafter, was chosen as the CV. WTMD–HREX was used to compute the free energy as a function of *z*. In the highest-order replica, the potential energy surface was modified to accelerate the dehydration of the five Ca^2+^ ions surrounding the vacancy and also to repel water from inside the vacancy.

We used LAMMPS,^24^ extended by our own implementation of WTMD–HREX, which we have released to the public.^25^ We validated our code using a toy model (see Supporting Information). The simulation was performed in the *NVT* ensemble with a 1 fs time step. The calcite slab was six layers thick and comprised a 5 × 3 surface supercell, and the water layer was approximately 30 Å thick (Figure S1). A temperature of 300 K was maintained by a chain of five Nosé–Hoover thermostats with a relaxation time of 0.1 ps. The interactions between CaCO_3_ and CaCO_3_–water were modeled using rigid ion potentials developed by Raiteri et al.,^4^ and the interactions between water molecules were described using the flexible SPC/Fw potential.^28^ Electrostatics were evaluated by using the P^3^ M method with an error tolerance of 10^–5^. A Ca^2+^ ion in the middle of the slab was left out of the integration procedure to keep it fixed in place and prevent the slab from drifting. The adsorbate position was confined by harmonic walls to a cuboid volume defined by the four Ca^2+^ ions in the surface layer surrounding the vacancy and extending 15 Å above the surface. The *z* coordinates of the five Ca^2+^ ions surrounding the vacancy were tethered to their average positions to prevent the vacancy from fragmenting.

Five HREX replicas were used with λ_*i*_ linearly interpolated from λ_1_ = 0 to λ_5_ = 1 and with exchange attempts every τ_swap_ = 1 ps. In replica 5, the interaction between the water and the five Ca^2+^ ions surrounding the vacancy was reduced by 40%. This resulted in sub-100 ps water exchange times for the four Ca^2+^ ions in the surface layer. Despite this weakening of the interaction, water remained in the vacancy for most of the simulation. An energy penalty was therefore added in the form of two Gaussians (see eq 7) with parameters *x*_1_^*^ = (0,0,–0.5 Å), *A*_1_ = 10 *k*_B_*T*, *B*_1_ = 1.5 Å and *x*_2_^*^ = (0,0,–2.3 Å), *A*_2_ = 14.8 *k*_B_*T*, *B*_2_ = 1 Å, where the origin coincided with the lattice site of the vacancy. The first Gaussian pushed the water out of the vacancy, while the second Gaussian ensured that water could not get trapped underneath the first Gaussian. This energy penalty maintained a dry vacancy in replica 5 throughout the simulation.

The bias potential was built from Gaussians of width σ = 0.1 Å and initial height *h* = *k*_B_*T* deposited every τ_deposit_ = 1 ps. Bias factors γ_*i*_ were linearly interpolated across the replicas from γ_1_ = 30 to γ_5_ = 200. Nine walkers were used per replica for a total of 45 simulations, which were run for an aggregate time of 1 μs (200 ns per replica).

To evaluate the effect of HREX, the simulation described above was repeated but with a single replica (equivalent to not using HREX) and 48 walkers.

### 3.2 Results

**Figure 2:**
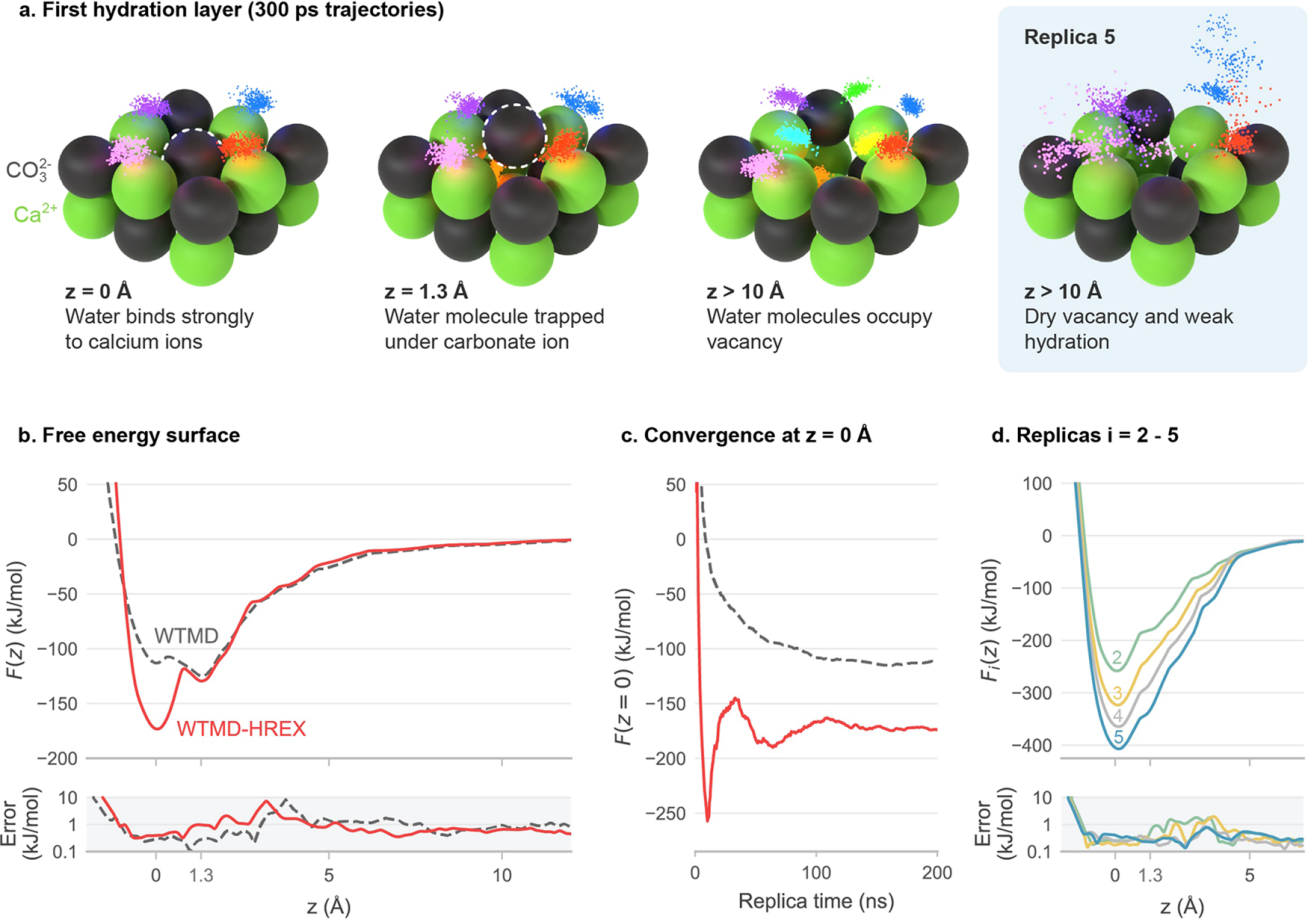
Formation free energy of a CO3^2–^ vacancy in the calcite–water interface. (a) Snapshots of the CO_3_^2–^ ion at different distances *z* from the surface, where the first three images are from replica 1 and the fourth from replica 5. The small dots represent the positions of oxygen atoms in the water over time, showing 300 ps long trajectories of water molecules initially coordinated with the Ca^2+^ ions, where each color corresponds to a distinct molecule. (b) Free energy surfaces obtained using WTMD–HREX (solid line) and WTMD without HREX (dashed line). Errors were computed using block analysis. (c) *F* evaluated at *z* = 0 Å versus simulation time, demonstrating convergence of the free energy surfaces. (d) Free energy surfaces for replicas 2 to 5.

The impact of the modified potential energy surface can be seen in the trajectories of water molecules initially coordinated with surface Ca^2+^ ions (Figure 2a). In replica 1, the water molecules remain bound to the Ca^2+^ ions for at least 300 ps, and they fill the vacancy when the adsorbate is dissolved (*z* > 10 Å). By contrast, in replica 5, the water molecules detach from the Ca^2+^ ions within 100 ps due to the weakened Ca^2+^–water bonds, and the vacancy remains free of water due to the energy penalty. The adsorbate can therefore enter the vacancy or adsorb to the Ca^2+^ ions in replica 5 without being impeded by water.

After sampling for 200 ns per replica, the free energy profiles *F*(*z*) obtained by using both WTMD–HREX and WTMD converged with fluctuations on the order of *k*_B_*T*. We averaged the free energy profiles, as shown in Figure 2b, over the final 40% of the simulation in blocks of 10 ns, using block analysis to quantify the errors.^2^Figure 2c shows *F* evaluated at *z* = 0 Å over the course of the simulation, demonstrating that the final 40% was a convergence phase. Figure 2d shows *F*_*i*_ for replicas *i* = 2 to 5, also averaged over the final 40% of the simulation as described above. The free energy profiles shown in Figure 2b–d are tabulated in Table S1.

While both WTMD–HREX and WTMD produced minima in *F* at *z* = 0 Å (corresponding to the lattice site) and at *z* = 1.3 Å (where the adsorbate binds to the vacancy with a water molecule trapped inside), they generated significantly different free energies. WTMD–HREX gave a formation free energy of 173.2 ± 0.6 kJ/mol, while WTMD without HREX indicated that the lattice site was metastable with a free energy that was only 113.2 ± 1.2 kJ/mol lower than that of the dissolved state. WTMD undersampled the lattice site because *z* = 0 Å could only be accessed by trapping a water molecule in an interstitial site. This interstitial water greatly reduced the stability of the lattice site; however, it did not impede access to the site, as reflected by the small errors near *z* = 0 Å in Figure 2b.

**Figure 3:**
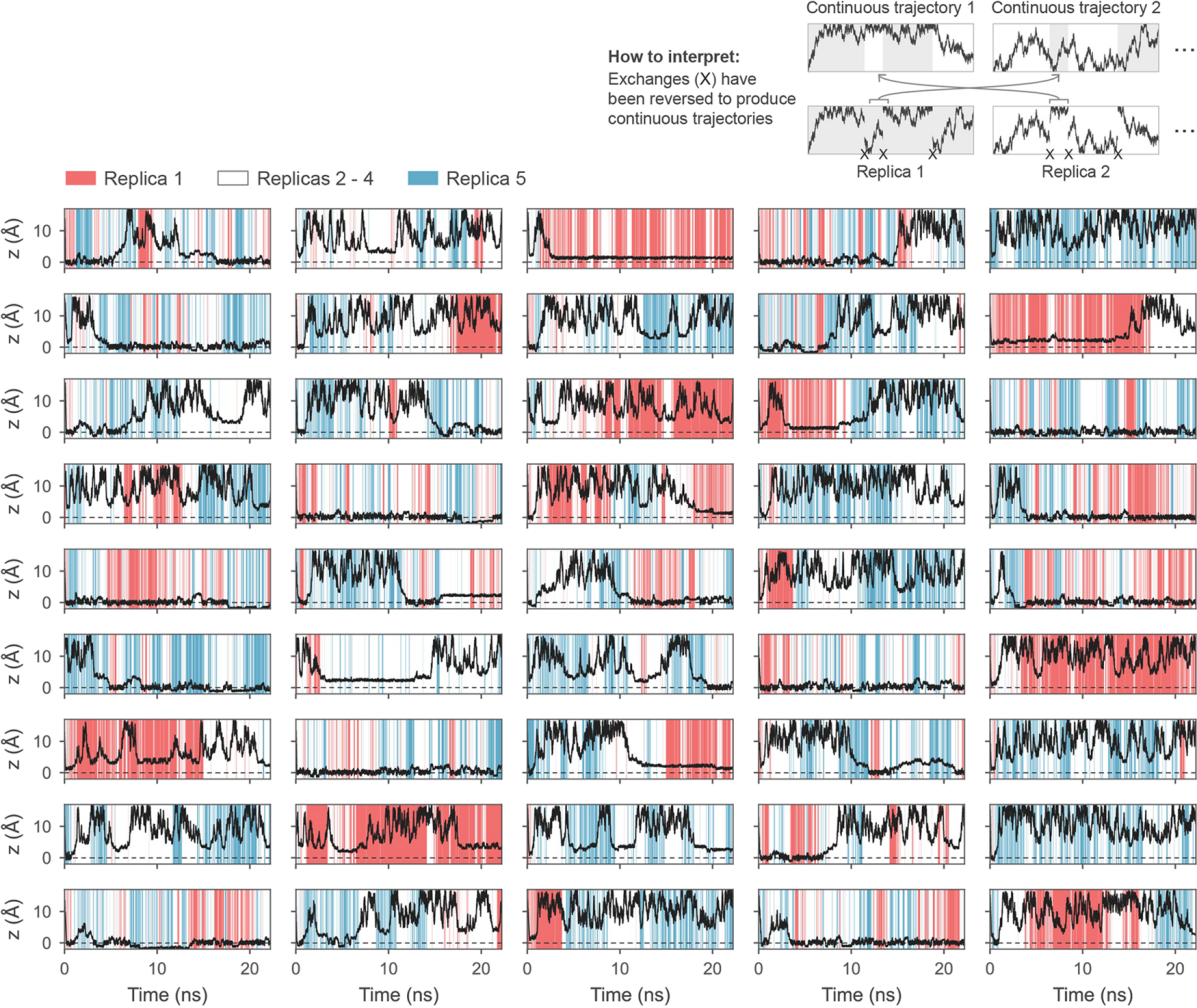
Sampling of the distance *z* between the CO_3_^2–^ ion and the calcite surface as the continuous trajectories randomly traverse replica space (represented by colors). Each of the nine rows corresponds to a separate WTMD walker. Transitions into and out of the *z* = 0 Å state demonstrate equilibrium sampling. Mixing between the replicas is most efficient when *z* = 0 Å, as explained in the main text.

To establish equilibrium sampling, it is not sufficient to observe that the barrier to enter the lattice site vanishes in the free energy surface of replica 5 (Figure 2d), since a barrier could remain orthogonal to *z*. Instead, an indicator of equilibrium sampling is that the continuous trajectories walking through the replica space display transitions between the various states in *z*. Indeed, most of the trajectories entered the lattice site at *z* = 0 Å within the first nanosecond of each simulation, and as the WTMD bias potential grew and increasingly compensated for the free energy surface, many of the trajectories left again (Figure 3). In the convergence phase (the final 40% of the simulation), there were six transitions away from *z* = 0 Å and six transitions into *z* = 0 Å. This balanced ingress and egress demonstrates that the trajectory population was free to evolve and likely attained equilibrium. Some of the continuous trajectories remained in the *z* = 0 Å state throughout the simulation because it was generally quicker for a replica to escape *z* = 0 Å by swapping configurations with a neighboring replica where *z* > 0 Å, rather than waiting for the configuration to diffuse away from *z* = 0 Å.

## 4 Discussion

In the preceding section, we demonstrated that the combination of WTMD with HREX can improve the free energy sampling for reactions involving slow solvent exchange. This approach has a wide-ranging scope since the functional form for Δ*U* presented in eq 6 is generally applicable to adsorption and complexation reactions in solution. The main task lies in determining suitable parameters, but we have described a straightforward procedure for this purpose (Section 2.3).

WTMD–HREX has two key advantages over the standard approach, where WTMD is used with an expanded CV space that features solute–solvent coordination numbers. First, when Δ*U* is appropriately chosen, WTMD–HREX can be applied to solutes with large and complex solvation shells. In contrast, it is unclear how reliable the standard approach is when there is more than one coordinating solvent molecule due to the non-Markovian dynamics of coordination numbers. Second, WTMD–HREX can efficiently handle the desolvation of a large number of atoms. Using WTMD to compute the free energy surface for one CV plus a single coordination number CV typically requires around 300 ns to converge.^29^ By comparison, our simulation, in which we accelerated solvent exchange for five ions, required a comparable simulation time of 1 μs. Furthermore, the cost of the WTMD approach scales exponentially with the number of atoms that require accelerated solvent exchange, whereas the number of replicas in WTMD–HREX scales only as the square root of this number.

In our application in Section 3, we focused on improving sampling in one particular region of CV space: the lattice site at *z* = 0 Å. However, it is important to exercise caution when applying WTMD–HREX to enhance sampling across multiple regions of the CV space, especially when using an energy penalty term. To illustrate why, consider Figure 3, where the quality of mixing between the first and last replicas can be discerned by the rate of alternation between red and blue bands of color. We observed good mixing between the first and last replicas when the adsorbate was located at *z* = 0 Å, but less efficient mixing when *z* > 0 Å. The reduced mixing for *z* > 0 Å arose because replica 1 favored a hydrated vacancy, while replica 5 favored a dehydrated vacancy due to the energy penalty. This problem is unlikely to arise if only the solute–solvent bonds are weakened with no energy penalty included since the generated configurations should be tolerated by all replicas. Otherwise, to improve sampling across multiple regions of the CV space, additional replicas may be needed, or the separate regions could be addressed independently.

## 5 Conclusions

Slow solvent exchange in molecular simulation poses a challenge to free energy methods by hindering the sampling of configurations. We have shown that sampling can be improved by combining the free energy method with HREX, along with a suitable potential energy modification to accelerate solvent exchange. This approach has broad applicability to adsorption and complexation reactions in solution, including reactions involving large molecules due to the efficient scaling of HREX.

## Supporting Information Available

Image of the full simulation cell; tabulated free energy data; and validation of our code using a toy model (PDF)

## References

[ref1] Demichelis R.; Schuitemaker A.; Garcia N. A.; Koziara K. B.; De La Pierre M.; Raiteri P.; Gale J. D. Simulation of crystallization of biominerals. Annu. Rev. Mater. Res. 2018, 48, 327–352. 10.1146/annurev-matsci-070317-124327.

[ref2] Bussi G.; Laio A. Using metadynamics to explore complex free-energy landscapes. Nat. Rev. Phys. 2020, 2, 200–212. 10.1038/s42254-020-0153-0.

[ref3] De La Pierre M.; Raiteri P.; Stack A. G.; Gale J. D. Uncovering the atomistic mechanism for calcite step growth. Angew. Chem., Int. Ed. 2017, 56, 8464–8467. 10.1002/anie.201701701. 28407395

[ref4] Raiteri P.; Demichelis R.; Gale J. D. Thermodynamically consistent force field for molecular dynamics simulations of alkaline-earth carbonates and their aqueous speciation. J. Phys. Chem. C 2015, 119, 24447–24458. 10.1021/acs.jpcc.5b07532.

[ref5] Liu L. M.; Laio A.; Michaelides A. Initial stages of salt crystal dissolution determined with ab initio molecular dynamics. Phys. Chem. Chem. Phys. 2011, 13, 13162–13166. 10.1039/c1cp21077g. 21637872

[ref6] Aufort J.; Schuitemaker A.; Green R.; Demichelis R.; Raiteri P.; Gale J. D. Determining the adsorption free energies of small organic molecules and intrinsic ions at the terrace and steps of calcite. Cryst. Growth Des. 2022, 22, 1445–1458. 10.1021/acs.cgd.1c01414.

[ref7] Armstrong B. I.; Silvestri A.; De La Pierre M.; Raiteri P.; Gale J. D. Determining the complete stability of calcite kink sites: Real vs ideal. J. Phys. Chem. C 2023, 127, 13958–13968. 10.1021/acs.jpcc.3c02864.

[ref8] Earl D. J.; Deem M. W. Parallel tempering: Theory, applications, and new perspectives. Phys. Chem. Chem. Phys. 2005, 7, 3910–3916. 10.1039/b509983h. 19810318

[ref9] Fukunishi H.; Watanabe O.; Takada S. On the Hamiltonian replica exchange method for efficient sampling of biomolecular systems: Application to protein structure prediction. J. Chem. Phys. 2002, 116, 9058–9067. 10.1063/1.1472510.

[ref10] Liao C.; Zhou J. Replica-exchange molecular dynamics simulation of basic fibroblast growth factor adsorption on hydroxyapatite. J. Phys. Chem. B 2014, 118, 5843–5852. 10.1021/jp501463r. 24815540

[ref11] Liu P.; Kim B.; Friesner R. A.; Berne B. J. Replica exchange with solute tempering: A method for sampling biological systems in explicit water. Proc. Natl. Acad. Sci. U.S.A. 2005, 102, 13749–13754. 10.1073/pnas.0506346102. 16172406 PMC1236566

[ref12] Wang L.; Friesner R. A.; Berne B. J. Replica exchange with solute scaling: a more efficient version of replica exchange with solute tempering (REST2). J. Phys. Chem. B 2011, 115, 9431–9438. 10.1021/jp204407d. 21714551 PMC3172817

[ref13] Bussi G.; Gervasio F. L.; Laio A.; Parrinello M. Free-energy landscape for β hairpin folding from combined parallel tempering and metadynamics. J. Am. Chem. Soc. 2006, 128, 13435–13441. 10.1021/ja062463w. 17031956

[ref14] Camilloni C.; Provasi D.; Tiana G.; Broglia R. A. Exploring the protein G helix free-energy surface by solute tempering metadynamics. Proteins: Struct., Funct., Genet. 2008, 71, 1647–1654. 10.1002/prot.21852. 18076039

[ref15] Prakash A.; Sprenger K. G.; Pfaendtner J. Essential slow degrees of freedom in protein-surface simulations: A metadynamics investigation. Biochem. Biophys. Res. Commun. 2018, 498, 274–281. 10.1016/j.bbrc.2017.07.066. 28720500

[ref16] Deighan M.; Bonomi M.; Pfaendtner J. Efficient simulation of explicitly solvated proteins in the well-tempered ensemble. J. Chem. Theory Comput. 2012, 8, 2189–2192. 10.1021/ct300297t. 26588950

[ref17] Li L.; Casalini T.; Arosio P.; Salvalaglio M. Modeling the structure and interactions of intrinsically disordered peptides with multiple replica, metadynamics-based sampling methods and force-field combinations. J. Chem. Theory Comput. 2022, 18, 1915–1928. 10.1021/acs.jctc.1c00889. 35174713 PMC9097291

[ref18] Zhai Y.; Laio A.; Tosatti E.; Gong X. G. Finite temperature properties of clusters by replica exchange metadynamics: the water nonamer. J. Am. Chem. Soc. 2011, 133, 2535–2540. 10.1021/ja1076316. 21291212

[ref19] Zhang Z.; Sponer J.; Bussi G.; Mlynsky V.; Sulc P.; Simmons C. R.; Stephanopoulos N.; Krepl M. Atomistic picture of opening–closing dynamics of DNA Holliday junction obtained by molecular simulations. J. Chem. Inf. Model. 2023, 63, 2794–2809. 10.1021/acs.jcim.3c00358. 37126365 PMC10170514

[ref20] Binette V.; Côté S.; Mousseau N. Free-energy landscape of the amino-terminal fragment of huntingtin in aqueous solution. Biophys. J. 2016, 110, 1075–1088. 10.1016/j.bpj.2016.01.015. 26958885 PMC4788745

[ref21] Raiteri P.; Laio A.; Gervasio F. L.; Micheletti C.; Parrinello M. Efficient reconstruction of complex free energy landscapes by multiple walkers metadynamics. J. Phys. Chem. B 2006, 110, 3533–3539. 10.1021/jp054359r. 16494409

[ref22] Barducci A.; Bussi G.; Parrinello M. Well-tempered metadynamics: a smoothly converging and tunable free-energy method. Phys. Rev. Lett. 2008, 100, 020603 10.1103/PhysRevLett.100.020603. 18232845

[ref23] Lingenheil M.; Denschlag R.; Mathias G.; Tavan P. Efficiency of exchange schemes in replica exchange. Chem. Phys. Lett. 2009, 478, 80–84. 10.1016/j.cplett.2009.07.039.

[ref24] Thompson A. P.; Aktulga H. M.; Berger R.; Bolintineanu D. S.; Brown W. M.; Crozier P. S.; in’t Veld P. J.; Kohlmeyer A.; Moore S. G.; Nguyen T.; Shan R.; Stevens M. J.; Tranchida J.; Trott C.; Plimpton S. J. LAMMPS-a flexible simulation tool for particle-based materials modeling at the atomic, meso, and continuum scales. Comput. Phys. Commun. 2022, 271, 108171 10.1016/j.cpc.2021.108171.

[ref25] Darkins R. HREX and WTMD-HREX for LAMMPS; GitHub Repository, 2023 https://github.com/RDarkins/lammps-hrex (accessed 18 Oct 2023).

[ref26] De La Pierre M.; Raiteri P.; Gale J. D. Structure and dynamics of water at step edges on the calcite {1014} surface. Cryst. Growth Des. 2016, 16, 5907–5914. 10.1021/acs.cgd.6b00957.

[ref27] Predescu C.; Predescu M.; Ciobanu C. V. On the efficiency of exchange in parallel tempering Monte Carlo simulations. J. Phys. Chem. B 2005, 109, 4189–4196. 10.1021/jp045073+. 16851481

[ref28] Wu Y.; Tepper H. L.; Voth G. A. Flexible simple point-charge water model with improved liquid-state properties. J. Chem. Phys. 2006, 124. 10.1063/1.2136877. 16422607

[ref29] Schuitemaker A. Computer Simulations of the Adsorption of Organic Molecules on Calcium Carbonate. Ph.D. Thesis; Curtin University, 2019;.

