# Supplementary material for: Accelerating Solvent Dynamics with Replica Exchange for Improved Free Energy Sampling
Source: J Chem Theory Comput. 2023 Oct 21;19(21):7527–32. doi: 10.1021/acs.jctc.3c00786 (PMC10653078; doi:10.1021/acs.jctc.3c00786)
Supplement: Supplementary file 1 — ct3c00786_si_001.pdf [file ct3c00786_si_001.pdf]

Supporting information for: Accelerating solvent dynamics with replica exchange for improved free energy sampling

Robert Darkins,* Dorothy M. Duffy, and Ian J. Ford

Department of Physics and Astronomy, University College London,
Gower Street, London, WC1E 6BT, UK

* r.darkins@ucl.ac.uk

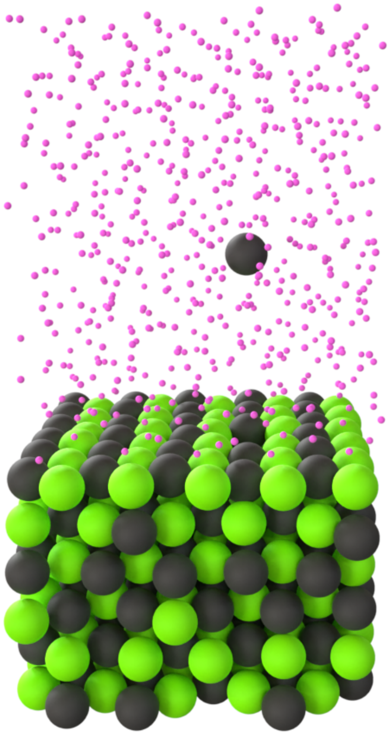

Figure S1: Snapshot of the full simulation cell showing calcium atoms (green), carbon atoms from the carbonate ions (grey) and oxygen atoms from the water molecules (pink). Hydrogen atoms from the water molecules and oxygen atoms from the carbonate ions are not shown for clarity.

z (Å)	Free energy (kJ/mol)					
	WTMD	WTMD-HREX replica				
		1	2	3	4	5
-1.0	-32.9	-17.0	-77.0	-125.8	-163.0	-200.0
-0.9	-48.5	-53.8	-116.2	-167.5	-205.8	-243.8
-0.8	-61.9	-86.2	-150.9	-202.6	-240.8	-280.3
-0.7	-74.0	-113.1	-181.3	-234.3	-269.5	-310.0
-0.6	-84.1	-132.9	-204.6	-260.1	-294.4	-334.8
-0.5	-92.3	-145.4	-220.8	-278.7	-313.9	-354.4
-0.4	-99.1	-154.0	-232.7	-292.4	-329.1	-370.1
-0.3	-104.5	-161.2	-242.5	-303.6	-341.7	-383.2
-0.2	-108.9	-167.2	-249.8	-312.5	-351.5	-393.1
-0.1	-111.9	-171.3	-255.2	-318.6	-358.6	-400.3
0.0	-113.2	-173.2	-257.9	-322.3	-363.0	-404.9
0.1	-112.4	-172.9	-258.1	-323.6	-364.7	-407.0
0.2	-110.0	-170.0	-256.1	-322.2	-364.1	-406.6
0.3	-107.6	-164.7	-251.2	-318.0	-361.0	-402.9
0.4	-107.4	-157.1	-244.2	-311.4	-354.6	-397.0
0.5	-108.6	-147.4	-234.6	-302.4	-345.8	-389.1
0.6	-110.4	-135.6	-223.6	-291.9	-335.6	-379.3
0.7	-112.1	-123.8	-211.3	-280.1	-324.2	-368.9
0.8	-114.3	-118.2	-198.6	-267.5	-312.7	-358.5
0.9	-116.3	-119.8	-188.5	-255.0	-302.4	-350.3
1.0	-118.5	-122.3	-184.8	-246.6	-296.8	-346.5
1.1	-120.6	-125.6	-182.9	-242.3	-293.1	-343.7
1.2	-123.3	-128.1	-181.2	-237.0	-288.5	-339.4
1.3	-124.6	-129.5	-180.2	-230.5	-281.4	-332.7
1.4	-124.5	-128.9	-178.4	-223.8	-272.9	-323.7
1.5	-122.3	-126.7	-175.6	-218.2	-262.9	-312.7
1.6	-118.7	-122.1	-170.8	-212.5	-253.4	-300.0
1.7	-113.1	-116.4	-164.4	-206.2	-244.8	-287.9
1.8	-107.3	-111.4	-157.4	-198.4	-235.6	-276.1
1.9	-102.6	-107.8	-151.8	-189.6	-225.1	-264.1
2.0	-99.5	-104.0	-147.6	-183.7	-215.8	-254.0
3.0	-56.9	-56.1	-80.5	-107.1	-140.4	-174.9
4.0	-41.3	-39.5	-49.4	-58.2	-67.7	-88.2
5.0	-25.9	-21.3	-27.6	-30.4	-29.9	-31.0
6.0	-14.8	-11.8	-15.0	-15.1	-14.7	-17.0
7.0	-11.1	-9.6	-10.6	-10.7	-9.3	-10.7
8.0	-7.6	-6.5	-7.4	-7.6	-5.9	-9.1
9.0	-5.7	-5.0	-5.4	-5.9	-4.6	-7.4
10.0	-4.4	-3.5	-3.6	-4.9	-3.3	-4.5
15.0	0.0	0.0	0.0	0.0	0.0	0.0

Table S1: Partial tabulated version of the free energy profiles obtained using WTMD and WTMD-HREX, as shown in Figure 2. The free energies are a function of the surface-carbonate distance z, and are reported relative to $z = 15$ Å. For WTMD-HREX, the free energies are included for all five replicas.

S1 Code validation

To validate our LAMMPS-based WTMD-HREX implementation, we developed a toy model that resembles the vacancy problem in the main paper, but that allows direct evaluation of the free energy surface. We were therefore able to compare the free energy surfaces obtained using WTMD and WTMD-HREX with the numerically exact result.

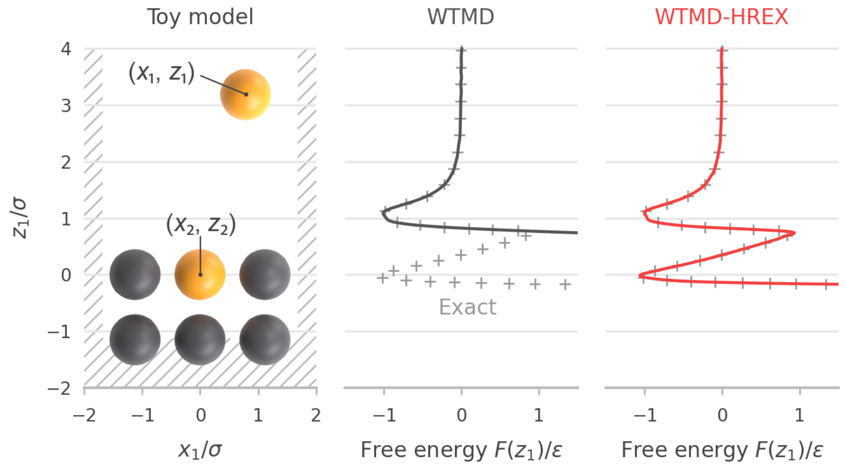

Figure S2: Toy model is two-dimensional and comprises seven particles: five are immobile (grey) and two are mobile (yellow). The mobile particles are confined by harmonic walls (hatching). Free energy as a function of z_1 is shown as obtained using WTMD (black) and WTMD-HREX (red). The numerically exact free energy is also shown (pluses).

S1.1 Toy model

The toy model comprises seven particles confined to the (x, z) plane (Figure S2). Two of the particles are mobile while the other five are fixed in a U-shape configuration analogous to a surface vacancy.

The two mobile particles have positions (x_1, z_1) and (x_2, z_2). Particle 1 represents an adsorbate for which we seek the free energy surface $F(z_1)$, while particle 2 mimics a solvent molecule in the sense that it competes with the adsorbate (particle 1) for access to the vacancy. The initial position of each particle is listed in Table S2 in terms of an arbitrary length-scale σ. The temperature of the system is $T = 0.1\epsilon/k_B$ where ϵ is an arbitrary energy scale and k_B is the Boltzmann constant.

Particle	Initial position		Mobile?
	x/σ	z/σ	
1	0	9	Yes
2	0	0	Yes
3	$-2^{1/6}$	0	No
4	$2^{1/6}$	0	No
5	$-2^{1/6}$	$-2^{1/6}$	No
6	0	$-2^{1/6}$	No
7	$2^{1/6}$	$-2^{1/6}$	No

Table S2: Initial position of each particle in the toy model.

Particles 1 and 2 are attracted to particles 3-7, while particles 1 and 2 repel each other. Since the system is non-periodic, particles 1 and 2 are restrained to the domain

$$\Omega = [-\frac{3}{2}2^{1/6}\sigma, \frac{3}{2}2^{1/6}\sigma] \times [-2^{1/6}\sigma, 10\sigma] \tag{1}$$

by harmonic walls.

To be more specific, the Hamiltonian is

$$\mathcal{H} = \sum_{i=1}^{2} \frac{1}{2} m(\dot{x}_i^2 + \dot{z}_i^2) + U \tag{2}$$

where m is the mass of particles 1 and 2 (the value of m is arbitrary), U is the potential energy surface

$$U = u_{\text{LJ}}^{\text{shift}}(r_{12}; 2^{1/6}\sigma) + \sum_{i=1}^{2}\sum_{j=3}^{7} u_{\text{LJ}}(r_{ij}) + \sum_{i=1}^{2} w(x_i, z_i), \tag{3}$$

r_{ij} is the distance between particles i and j, u_{LJ} is the Lennard-Jones potential

$$u_{\text{LJ}}(r) = 4\epsilon \left[\left(\frac{\sigma}{r}\right)^{12} - \left(\frac{\sigma}{r}\right)^{6} \right], \tag{4}$$

$u_{\text{LJ}}^{\text{shift}}$ is the shifted cut-off version

$$u_{\text{LJ}}^{\text{shift}}(r; r_c) = \begin{cases} u_{\text{LJ}}(r) - u_{\text{LJ}}(r_c) & r < r_c \\ 0 & r \geq r_c \end{cases} \tag{5}$$

and finally w is the harmonic wall

$$w(x_i, z_i) = 100\epsilon(\Delta x_i^2 + \Delta z_i^2) \tag{6}$$

where $(\Delta x_i, \Delta z_i)$ equals $(0, 0)$ if (x_i, z_i) is inside the domain Ω, otherwise $(\Delta x_i, \Delta z_i)$ is the displacement between (x_i, z_i) and the nearest point on the boundary of Ω.

Note that the cutoff choice $r_c = 2^{1/6}\sigma$ in the shifted Lennard-Jones potential in equation (3) yields a purely repulsive interaction.

S1.2 Exact free energy surface

Since there are only four degrees of freedom, the free energy surface,

$$F(z_1) = -k_B T \log \int \exp(-U(x_1, z_1, x_2, z_2)/k_B T)\, \mathrm{d}x_1\, \mathrm{d}x_2\, \mathrm{d}z_2, \tag{7}$$

can be evaluated to arbitrary precision using numerical integration, the results of which are represented by pluses in Figure S2.

S1.3 WTMD

We simulated the dynamics of the toy model using the Langevin equation with a damping parameter equal to $\sqrt{m\sigma^2/\epsilon}$ and an integration time step $\Delta t = 10^{-2}\sqrt{m\sigma^2/\epsilon}$, as implemented in LAMMPS.

To evaluate $F(z_1)$ we performed WTMD using our LAMMPS package. The collective variable was z_1. The Gaussians had a standard deviation of 0.05σ and they were deposited at intervals of $100\Delta t$. The Gaussian height was initially 0.1ϵ, but this decreased over time due to well-tempering (bias factor $\gamma = 10$). The simulation ran for 10^6 time steps.

The resulting free energy surface is shown in Figure S2. It can be seen that particle 1 was unable to access the vacancy at any point during the simulation due to particle 2 occupying the vacancy throughout. The free energy is therefore near-exact above the vacancy but erroneous near $z_1 \approx 0$.

S1.4 WTMD-HREX

The simulation described in the preceding section was repeated but with HREX enabled. Three replicas were used (replicas 1, 2 and 3). In replica 3, the interactions between particle 2 and particles 3-7 were reduced by 90%. In replica 2, the same interactions were reduced by 45%. In replica 1, the interactions were unchanged. Configurations were swapped between the replicas every $\tau_{\mathrm{swap}} = 10^3 \Delta t$.

These changes meant that in replica 3 (and to a lesser extent in replica 2), particle 2 could thermally hop out of the vacancy on a short time scale and explore the entire space, granting particle 1 access to the vacancy. Swaps between the replicas meant that the ergodicity was improved for all replicas.

The free energy surface $F(z_1)$ obtained using this method is shown in Figure S2. It is indistinguishable from the exact result, thus validating both the method and our implementation of it.
